# High-Uniformity Flat-Top Light Spot Based on a Dielectric Metasurface

**DOI:** 10.3390/nano16030208

**Published:** 2026-02-05

**Authors:** Xinxin Pu, Wenhao Guo, Jinyao Hou, Yechuan Zhu, Xueping Sun, Shun Zhou, Weiguo Liu

**Affiliations:** Shaanxi Province Key Laboratory of Thin Films Technology and Optical Test, Xi’an Techonlogical University, Xi’an 710021, China; pxx_1125@126.com (X.P.);

**Keywords:** metasurface, laser beam shaping, flat-top beams, phase control

## Abstract

With the rapid development of laser processing and infrared imaging, the demand for flat-top beams with high uniformity has become increasingly urgent. Conventional beam-shaping techniques based on bonded aspheric lenses are inherently bulky and inflexible, which limits their compatibility with modern optical systems. In this work, we propose a dielectric metasurface for laser beam shaping operating at 1064 nm, where the target phase distribution is derived by the given initial phase and is represented by a hyperbolic phase. An inverse optimization algorithm is proposed to optimize the unit cell consisting of silicon carbide (SiC) nanopillars and the silicon dioxide (SiO_2_) substrate. Numerical results show that, after transmission through the designed metasurface, the beam forms a circular flat-top spot with a radius of 2 μm at the target plane, exhibiting an intensity uniformity of 0.1021 and an energy efficiency of 76.3%. This study offers a compact and highly efficient solution for the flat-top beam shaping, demonstrating significant potential for applications in precision-laser processing, optical trapping, and bioimaging.

## 1. Introduction

The rapid development of modern optical technologies has imposed increasingly stringent requirements on the accuracy and customization of the spatial intensity distribution of laser beams [1,2]. For instance, in laser processing, the non-uniform intensity distribution of conventional Gaussian beams with higher intensity at the center and lower intensity at the edges results in steep temperature gradients, which in turn degrade the mechanical properties of the processed parts [3]. In the fields of precision lithography and biological imaging, the beam uniformity plays a critical role in determining processing resolution and imaging quality [4,5]. Diffractive optical elements (DOE) have the advantages of compact structure and flexible design. However, their diffraction mechanism leads to significant energy loss. The diffraction efficiency of typical DOE devices is generally lower than 60% [6,7,8]. Moreover, due to the limitation of phase quantization accuracy, the uniformity error of the generated flat-top light spot is usually greater than 0.15, making it difficult to meet the requirements of high-precision light-field control [9,10].

Metasurface possess advantages such as the phase degree of freedom and ease of integration, providing a new approach to overcoming the technical bottlenecks of beam shaping [11,12,13]. Previous studies have designed the phase distribution of the metasurface based on the Gerchberg–Saxton (GS) algorithm to realize the transformation from Gaussian beams to flat-top beams. However, this method is limited by the local convergence characteristics of the GS algorithm, which often leads to poor intensity uniformity and pronounced sidelobes [14,15,16]. Chen et al. designed anisotropic nanostructures based on the Pancharatnam–Berry (PB) phase principle, to achieve flat-top beam generation under high numerical aperture conditions. However, this approach is sensitive to the polarization state of the incident light, which results in low energy efficiency [17]. Wang et al. successfully achieved a collimated flat-top beam using a complex amplitude constrained GS algorithm based on cascaded metasurfaces [18]. However, the cascaded structure imposed challenges such as high fabrication complexity and stringent alignment accuracy requirements, and it was not optimized for the industrially common 1064 nm wavelength [19,20,21,22,23,24]. Despite these previously reported works, existing approaches still suffer from inherent limitations in designing homogenized focal spots that meet the requirements of complex applications when using either method alone [25,26,27,28,29,30,31].

Here, we report a dielectric metasurface operating at a wavelength of 1064 nm, which provides a novel and effective approach for laser beam shaping and focal spot homogenization. First, we obtain the target phase distribution by using the hyperbolic phase as the initial phase distribution. Then, we perform global optimization of the nanostructure parameters using an inverse optimization algorithm. Subsequently, we use the finite-difference time-domain (FDTD) method to simulate the interaction between light and unit cells to achieve precise control of the unit cell. Finally, we establish a correlation between the phase distribution and unit cells of metasurface, while clarifying the influence of the unit cells on laser-beam shaping.

## 2. Design and Methods

**Figure 1:**
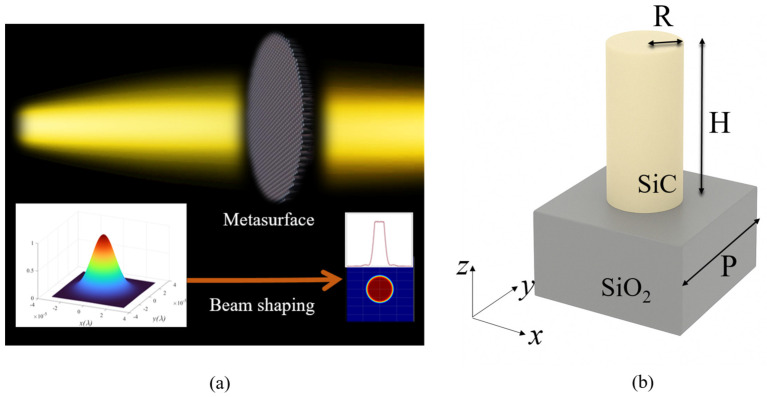
(**a**) A schematic of the laser-shaping metasurface. (**b**) The design of the phase-modulating unit cell is a figure.

The flat-top beam is generally achieved by precisely controlling the phase of the incident beam through optical elements, resulting in uniform light intensity distribution on the target plane. Figure 1a illustrates a schematic diagram of beam shaping by the metasurface. In order to obtain an ideal flat-top light intensity distribution on the target plane, a specific phase distribution needs to be designed and implemented using a metasurface. After phase modulation, the resulting optical field closely matches the target intensity distribution. In this study, the unit cell consisted of SiC nanopillars and the SiO_2_ substrate, as shown in Figure 1b. Here, R and H represent the radius and heights of the SiC nanopillars, respectively. The period of the unit cell is denoted by P. The phase varies with the H, R, and P of the unit cell. In this work, the phase varies with the radius of the unit cell when the H and P remained constant, enabling the generation of a phase delay distribution corresponding to the unit cells of different radii.

**Figure 2:**
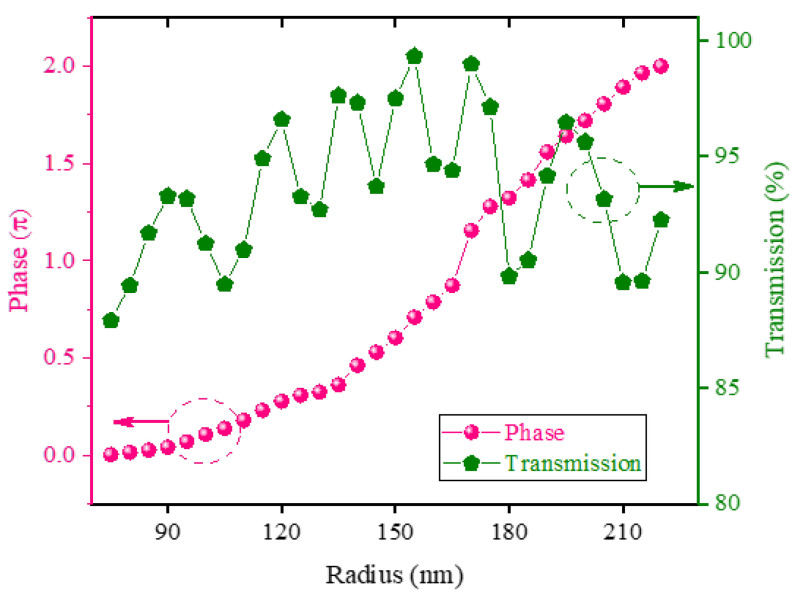
The plots of radius, phase and T of SiC nanopillars with *P* = 500 nm, *H* = 1000 nm.

Here, we use the FDTD method to simulate the phase response of the unit cell and analyze the constructed phase control element. The height of the SiC nanopillars was set to H = 1000 nm and the period of *P* = 500 nm to ensure an effective phase control. The phase shifts and transmittance spectra of SiC nanopillars are simulated across a range of radius, with the results presented in Figure 2. It is observed that a full 0~2π phase coverage is achieved when the radius of SiC nanopillars varies from 75 nm to 220 nm at a period of *P* = 500 nm. The transmittance exceeded 93% within the corresponding radius range. By optimizing the radius of the unit cell, we get an optimal unit cell that achieves full 0~2π phase coverage with high transmittance.

To solve the issues of poor uniformity and high sidelobe energy caused by the random initial phase selection in the conventional GS algorithms, this study proposes employing hyperbolic phases as the initial phase distribution. This guides the optimization process toward rapid convergence to high-performance solutions. Combined with an inverse optimization algorithm for global optimization, this approach enables an efficient control of the wavefront phase. The hyperbolic phase distribution is expressed as Equation (1):

(1) $$\varphi \left(x,y\right)=k\sqrt{x^{2}+y^{2}+f^{2}}+C\left(x^{2}+y^{2}\right)$$

where k=2π/λ is the wave number. f represents the focal length, and *C* represents the phase coefficient of hyperbolic. This phase distribution redistributes the optical intensity through nonlinear modulation, thereby promoting a more uniform intensity distribution in the central area of the target plane.

From a phase-modulation perspective, the phase profile of a conventional metalens is typically expressed as Equation (2):

(2) $$\phi_{\mathrm{Metalens}}(r)=-k\left(\sqrt{r^{2}+f^{2}}-f\right)$$

It can be observed that the conventional metalens phase profile is derived from the equal optical path length condition. As a result of this phase modulation, the incident light is focused on the target position, leading to a Gaussian-like intensity distribution in the focal plane.

In contrast, the hyperbolic phase profile adopted in this work introduces a customized phase modulation scheme to reconfigure the phase distribution over the entire aperture. This facilitates the spatial redistribution of optical intensity, leading to a homogeneous intensity distribution within the central region of the target plane. The transition from conventional point focusing to flat-top area intensity distribution thereby lays the physical foundation for generating a high-uniformity flat-top light spot.

**Figure 3:**
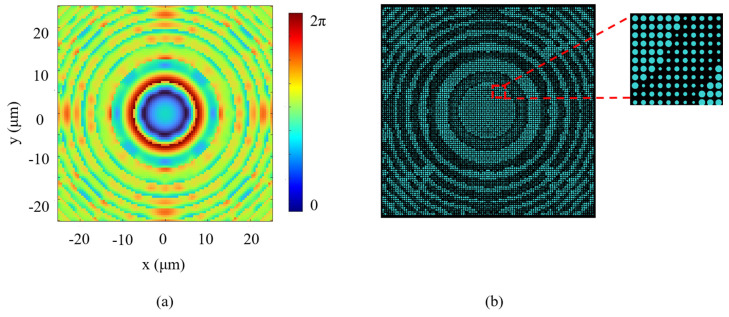
(**a**) The target phase distribution of the metasurface. (**b**) A schematic of the metasurface structural arrangement.

To improve the uniformity of the light spot and suppress stray light, an inverse optimization algorithm is employed to optimize the initial phase distribution. A target function is formulated using Equation (3), which aims to minimize the intensity uniformity error on the target plane while maximizing energy utilization efficiency:

(3) $$F=a\cdot \frac{\sigma \left(I\right)}{\bar{I}}+b\cdot \left(1-\frac{P_{flat}}{P_{in}}\right)$$

where $\sigma \left(I\right)$ and $\bar{I}$ denote the standard deviation and the average value of the intensity in the target plane, respectively. $P_{flat}$ represents the energy in the flat-top region, $P_{in}$ denotes the total incident energy, and *a* and *b* are weighting coefficients. The parameters of the nanopillars were inversely optimized based on the obtained target phase distribution until the objective function converges $\left\|\nabla F\right\|\leq 10^{-5}$. Finally, a metasurface is constructed based on the optimized target parameters. Within the 50 × 50 μm area of metasurface, the individual unit cells are arranged according to the phase distribution required for flat-top beam shaping. Figure 3a and Figure 3b show the optimal phase distribution and the arrangement with unit cells of the designed metasurface, respectively.

**Figure 4:**
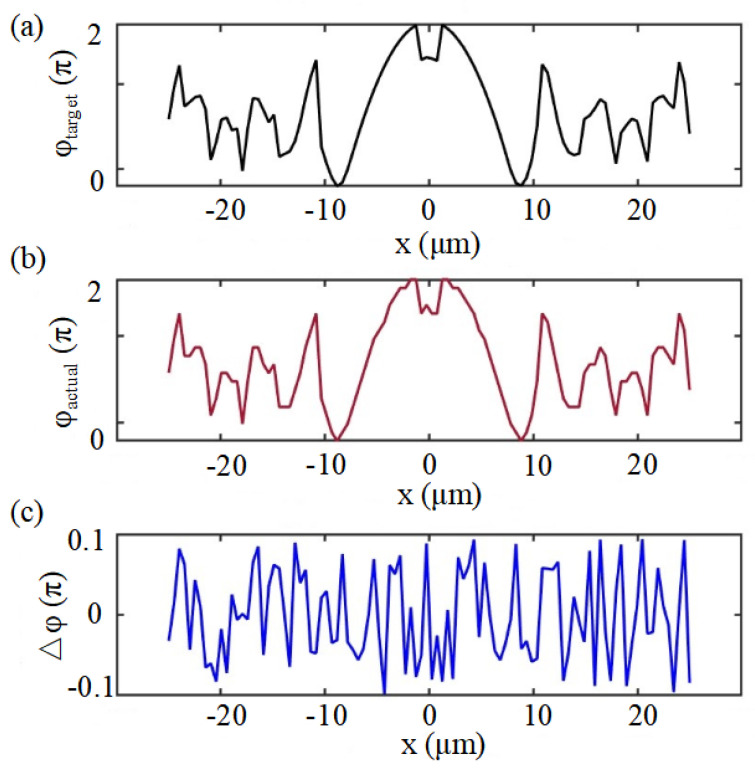
(**a**) Target phase distribution. (**b**) Achievable phase distribution obtained from the unitcell mapping. (**c**) Phase error between the achievable phase and the target phase.

Based on the optimized two-dimensional phase distribution shown in Figure 3, it is necessary to further examine whether the designed target phase can be faithfully implemented by the metasurface unit-cell mapping. To this end, Figure 4 presents a one-dimensional comparison between the target phase distribution and the achievable phase distribution extracted along the central cross-section of the metasurface aperture. As shown in Figure 4a,b, the achievable phase closely follows the target phase over the entire aperture. The corresponding phase error, plotted in Figure 4c, remains confined within a small range, indicating that the designed phase profile can be accurately realized in practice.

## 3. Simulation and Discussion

In this design, the metasurface diameter is initially set to 50 μm, and the incident wavelength is 1064 nm. At the operating wavelength, the refractive indices are approximately n_SiC_ ≈ 2.60 and n_SiO2_ ≈ 1.449, respectively. The parameter distribution of the nanopillars is obtained using an inverse optimization algorithm based on the target phase. Through numerical simulations, we analyzed the homogenization of the focal spots formed by the metasurface.

**Figure 5:**
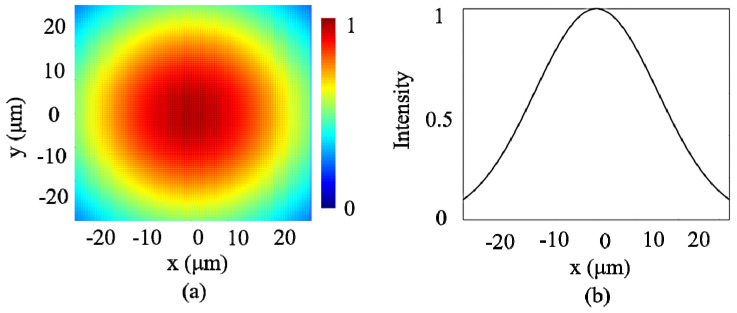
(**a**) The distribution of the incident optical field. (**b**) The intensity of the incident light field.

Figure 5 shows the distribution of the incident optical field. As shown in Figure 5a, the incident light is a collimated Gaussian beam whose intensity is truncated at the metasurface plane. The intensity is distributed according to a Gaussian pattern as shown in Figure 5b, with the beam waist radius w_0_ = 30 μm, ensuring the incident field covers the working area of the metasurface.

**Figure 6:**
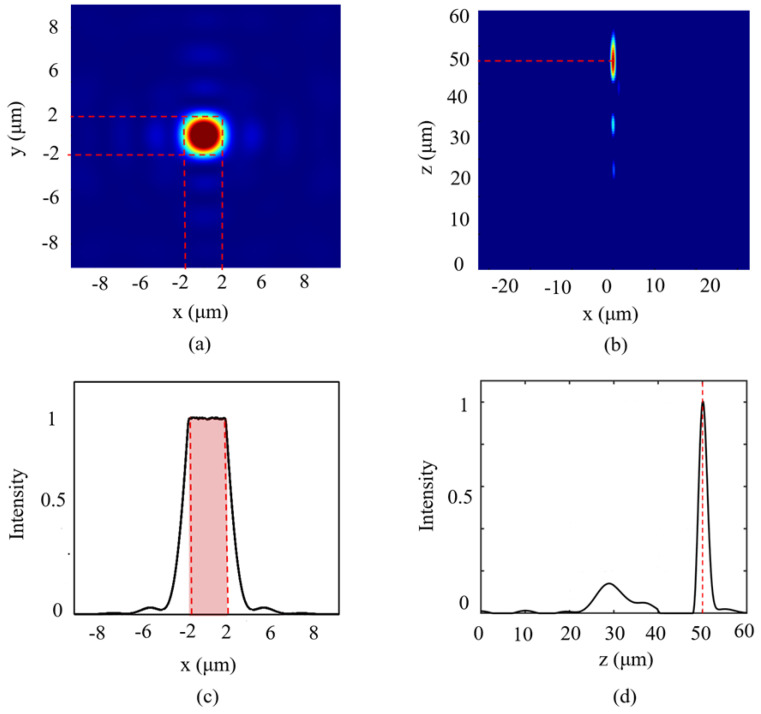
(**a**) Optical field distribution in the *x*-*y* plane. (**b**) Optical field distribution in the *x*-*z* plane. (**c**) Intensity distribution along the *x*-axis. (**d**) Intensity distribution along the *z*-axis.

After transmission through the designed metasurface, Figure 6a shows the two-dimensional intensity distribution of the 1064 nm beam on the *x*-*y* plane at the preset target plane. Figure 6b and Figure 6d display the two-dimensional intensity distribution on the *x*-*z* plane and the intensity distribution cross-section along the *z*-axis, respectively. It can be observed that the outgoing light forms a circular spot with clear, regular-shaped and concentrated energy on the target plane. Moreover, the position of the flat-top spot is consistent with the designed target plane at *z* = 50 μm. This preliminary verification demonstrates that the designed metasurface has the ability to shape the wavefront effectively. Figure 6c shows the intensity distribution curve along the center of the spot. This curve exhibits a typical flat-top profile. Within the central region of the spot at *r* = 2 μm, the intensity variation is minimal, indicating a highly uniform distribution. The size of the flat-top region aligns with the design target. This demonstrates that the metasurface successfully achieves the function of predefined customized wavefront shaping.

To evaluate the uniformity of the light spot, this study uses the standard deviation of all light intensity sampling points within the flat-top area as the uniformity metric. Based on the calculated results, the uniformity of the flat-top spot reaches 0.1021, indicating a highly consistent intensity distribution. Here, we define the efficiency of the metasurface as the ratio of the total optical energy within the flat-top region to the total incident optical energy. The results show that the designed metasurface achieves an energy utilization rate of 76.3% at a wavelength of 1064 nm. This result is attributed to the combined effect of two factors. Firstly, the average transmittance of the unit cells within the 2π phase coverage range is as high as 93%. Secondly, the optimized phase distribution effectively suppresses diffraction loss and stray light by importing hyperbolic phase as the initial phase distribution, thereby significantly improving the effective utilization rate of energy. Compared with conventional DOEs, the performance of the designed metasurface in this study has been improved. The uniformity error of the flat-top spot has decreased by 0.0479, which represents a relative increase of 31.93%, and the energy utilization rate has increased by 27.2%.

To further place the proposed metasurface in the context of existing flat-top beam-shaping approaches, Table 1 provides an advanced comparison of representative methods, including different methods, design strategies, and performance such as uniformity, efficiency, and side-lobe suppression ratio.

Based on the above comparison, it can be seen that the proposed single-layer, polarization-independent metasurface based on hyperbolic phase initialization and inverse optimization achieves a uniformity error of 0.1021, an efficiency of 76.3%, and a side-lobe suppression ratio of 8.39 dB at 1064 nm, demonstrating a well-balanced trade-off between optical performance and practical fabrication feasibility.

**Figure 7:**
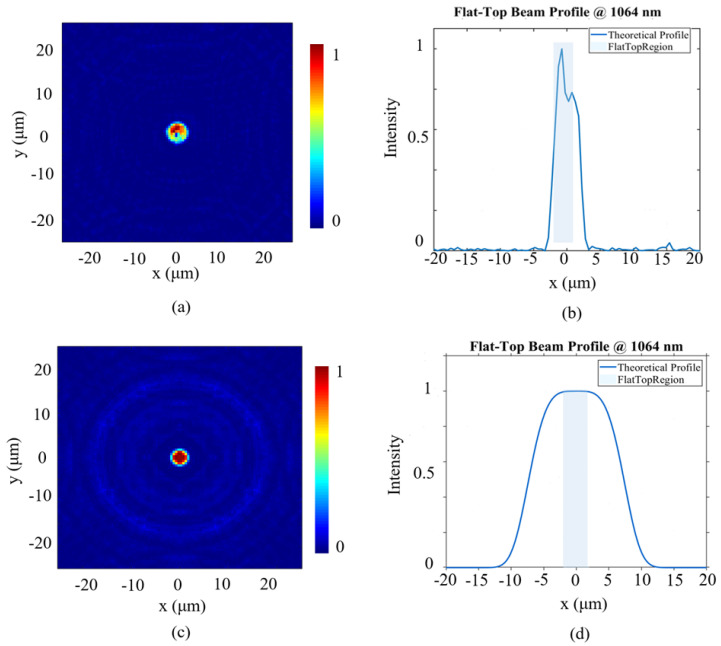
(**a**) Optical field distribution in the *x*-*y* plane for the metasurface designed with a conventional metalens phase profile. (**b**) Intensity distribution along the *x*-axis for the conventional metalens-based metasurface. (**c**) Optical field in the *x*-*y* plane for the metasurface designed with the proposed hyperbolic phase profile. (**d**) Intensity distribution along the *x*-axis with hyperbolic phase.

To evaluate the critical role of hyperbolic phase distribution in metasurface for the flat-top beam shaping, this study developed a comparative analysis framework. A metasurface design without hyperbolic phase initialization is used as the reference baseline, and the optical field modulation performance of the two schemes is compared through numerical simulations. The simulation results are shown in Figure 7.

Figure 7a and Figure 7c, respectively, show the distribution of the outgoing light intensity in the *x*-*y* plane for the metasurface without and with the introduction of the hyperbolic phase distribution. Figure 7b,d present the light intensity distributions along the *x*-axis for the two cases. Numerical simulation results show that, without importing the hyperbolic phase distribution, the intensity of the outgoing light field fluctuates significantly within the target area, and the uniformity is poor. A flat-top distribution that meets the requirements could not be formed. This indicates that the traditional phase distribution method has limitations in terms of the accuracy and stability of wavefront shaping. After importing a hyperbolic phase as the initial phase distribution, the light field rapidly converges into a flat-top spot with uniform intensity distribution and steep edges on the target plane. Its overall shape is in good agreement with the design goal. This comparison fully validates the critical role of hyperbolic phase distribution in achieving high-uniformity flat-top beam generation.

In summary, this study designed a metasurface based on a hyperbolic phase distribution at a working wavelength of 1064 nm and successfully achieved the generation and control of a flat-top beam. This research provides an effective wavefront control solution for advanced optoelectronic systems such as high-precision laser processing and optical trapping.

## 4. Conclusions

In conclusion, we demonstrate a dielectric metasurface with a target phase distribution designed by optimizing to achieve a homogenized focal spot at an incident wavelength of 1064 nm. By importing the hyperbolic phase as the initial distribution and combined with the inverse optimization algorithm, the phase and radius of the nanopillars for beam shaping were successfully designed. The designed metasurface generates a flat-top beam with a uniformity error of 0.1021 and an energy utilization rate of 76.3% at the target plane. Compared with traditional diffraction optical elements, the uniformity has improved by 31.93% and the energy utilization rate has increased by 27.2%. This work provides demonstration that the dielectric metasurface can achieve intensity-homogenized focal spots. Furthermore, the proposed approach is adaptable to high-performance, complex light-field regulation.

## Figures and Tables

**Table 1:** The comparison of flat-top beam-shaping methods.

Method	λ (nm)	Structure Type	Design Strategy	Uniformity	Efficiency (%)	SLSR (dB)
GS/IFTA-based phase metasurface	532/633	Single-layer, phase-only	Gerchberg–Saxton/IFTA	>0.15	50–65	~5–7
PB-phase metasurface	633	Single-layer, anisotropic	Pancharatnam–Berry phase	~0.12–0.15	<60	~6–8
Cascaded metasurface	532	Cascaded multi-layer	Complex-amplitude GS	<0.10	~70	>10
Multi-layer inverse-designed metasurface	Visible	Multi-layer dielectric	Inverse/hybrid optimization	<0.10	70–80	12–15
Diffractive optical element (DOE)	1064	Surface-relief element	Phase quantization	>0.15	<60	~5
This work	1064	Single-layer dielectric metasurface	Hyperbolic phase initialization + inverse optimization	0.1021	76.3	8.39

## Data Availability

Data are contained within the article. The data presented in this study are available in Section 2 (Design and Method) and Section 3 (Simulation and Discussion).
